# CEP164 Deficiency Causes Hyperproliferation of Pancreatic Cancer Cells

**DOI:** 10.3389/fcell.2020.587691

**Published:** 2020-11-05

**Authors:** Tetsuo Kobayashi, Kosuke Tanaka, Yu Mashima, Ayano Shoda, Mio Tokuda, Hiroshi Itoh

**Affiliations:** Division of Biological Science, Graduate School of Science and Technology, Nara Institute of Science and Technology, Ikoma, Japan

**Keywords:** CEP164, primary cilia, proliferation, hedgehog, PDAC

## Abstract

Primary cilia are hair-like projections that protrude from most mammalian cells and mediate various extracellular signaling pathways. Pancreatic ductal adenocarcinoma (PDAC) cells are known to lose their primary cilia, but the relevance of this phenomenon remains unclear. In this study, we generated PDAC-originated Panc1 cells devoid of primary cilia by mutating a centriolar protein, centrosomal protein 164 (CEP164), which is required for ciliogenesis. CEP164 depletion enhanced the clonogenicity of Panc1 cells, along with chemically induced elimination of primary cilia, suggesting that a lack of these organelles promotes PDAC cells proliferation. In addition, the loss of CEP164 altered the cell cycle progression irrespective of absence of primary cilia. We found that CEP164 was co-localized with the GLI2 transcription factor at the mother centriole and controlled its activation, thus inducing Cyclin D-CDK6 expression. Furthermore, CEP164-mutated Panc1 cells were significantly tolerant to KRAS depletion-dependent growth inhibition. This study suggests that CEP164 deficiency is advantageous for PDAC cells proliferation due to not only lack of ciliation but also cilia-independent GLI2-Cyclin D/CDK6 activation, and that CEP164 is a potential therapeutic target for PDAC.

## Introduction

Most human cells express a hair-like projection on their surface, referred to as primary cilium (Ishikawa and Marshall, 2011). As this organelle houses multiple signaling molecules that receive extracellular stimuli and transduce them into the cell body, it is considered to be the cells’ sensor. Numerous oncogenic signaling pathways such as the Hedgehog (Hh) and Wnt pathways are also processed through primary cilium (Eguether and Hahne, 2018; Liu et al., 2018).

The primary cilium extends from the centrioles, a pair of cylinder-like structures in the centrosome which plays a pivotal role in mitotic spindle formation (Kobayashi and Dynlacht, 2011). The older mother centriole is equipped with distal and subdistal appendages (DA and SDA) that are absent from the younger daughter centriole, and serves as the base of the primary cilium during interphase. Typically, serum deprivation of cultured mammalian cells triggers primary cilium assembly; small vesicles initially attach onto the DA, then an enlarged vesicle (the ciliary vesicle) covers the top of the mother centriole, developing into the ciliary membrane and encapsulating the ciliary shaft (Wang and Dynlacht, 2018). Therefore, proteins comprising the DA play an essential role in the formation of ciliary vesicles and subsequently the primary cilium (Schmidt et al., 2012; Joo et al., 2013; Ye et al., 2014). To date, several studies have reported that the ablation of DA proteins including centrosomal protein 164 (CEP164) causes severe loss of primary cilia (designated as “de-ciliation”) in cultured mammalian cells (Graser et al., 2007; Schmidt et al., 2012; Tanos et al., 2013).

Primary cilia are absent or reduced in multiple cancers, including pancreatic ductal adenocarcinoma (PDAC) (Eguether and Hahne, 2018). PDAC accounts for >90% of pancreatic cancer and its 5-year survival rate is only approximately 5–7% (Adamska et al., 2017). In most PDAC cases, the proto-oncogene, Kirsten rat sarcoma viral oncogene homolog (KRAS), is mutated to its constitutively active form. A previous study has demonstrated that KRAS signaling contributes to the suppression of primary ciliogenesis in PDAC cells (Seeley et al., 2009). We recently proposed that in addition to KRAS, histone deacetylase 2 (HDAC2) inhibits ciliation by regulating the expression of Aurora A kinase (AurA), which promotes the disassembly of the cilia in PDAC cells (Kobayashi and Itoh, 2017; Kobayashi et al., 2017).

Since primary cilia are involved in various oncogenic signaling pathways and cell cycle progression, lack of these organelles can influence the onset and/or progression of PDAC. In support of this hypothesis, KRAS- or HDAC2-dependent loss of primary cilia hastens cell cycle progression in PDAC cells (Kobayashi et al., 2017). A recent study has demonstrated that experimental diminution of primary cilia in normal pancreatic ductal cells promotes their transformation into cancer cells through potentiating the mevalonate (MVA) pathway, which is associated with increased cholesterol biosynthesis (Deng et al., 2018). However, disruption of ciliogenesis has been shown to both positively and negatively influence tumor growth, depending on the cancer type (Han et al., 2009; Wong et al., 2009; Gradilone et al., 2013; Ho et al., 2013; Hassounah et al., 2017; Yang et al., 2017; Lee et al., 2019).

In the present study, we generated PDAC-originated Panc1 cells devoid of primary cilia by mutating the DA protein CEP164 to elucidate the effect of de-ciliation on the proliferation of PDAC cells. We focused on CEP164 among many ciliary genes because low expression of CEP164 is associated with poor prognosis of pancreatic cancer patients (Uhlen et al., 2017). CEP164-mutated (Cep164-1) Panc1 cells exhibited increased colony formation. Chemical exclusion of primary cilia using chloral hydrate (ClHy) exerted a similar effect, suggesting that de-ciliation enhances the clonogenicity of Panc1 cells. Besides, Cep164-1 cells showed cell cycle alteration in serum-fed culture condition in which primary cilia are rarely formed, most likely due to augmented expression of Cyclin D-Cyclin dependent kinase 6 (CDK6), whereas ClHy-treated Panc1 cells did not exhibit similar phenotypes. These results suggest that CEP164 depletion causes these anomalies independently of de-ciliation. Importantly, CEP164 co-localized with GLI family zinc finger 2 (GLI2), a transcription factor of the Hh pathway, at the mother centriole, and loss of CEP164 abolished GLI2 foci and induced GLI2 activation, leading to Cyclin D-CDK6 expression. Furthermore, Cep164-1 cells were more resistant to growth retardation provoked by KRAS depletion than wild-type (WT) cells. Collectively, we propose that CEP164 deficiency provokes de-ciliation and cilia-independent activation of GLI2-Cyclin D/CDK6 axis, thereby leading to PDAC proliferation.

## Results

### Generation of CEP164-Mutated Panc1 Cells Devoid of Primary Cilia

**FIGURE 1:**
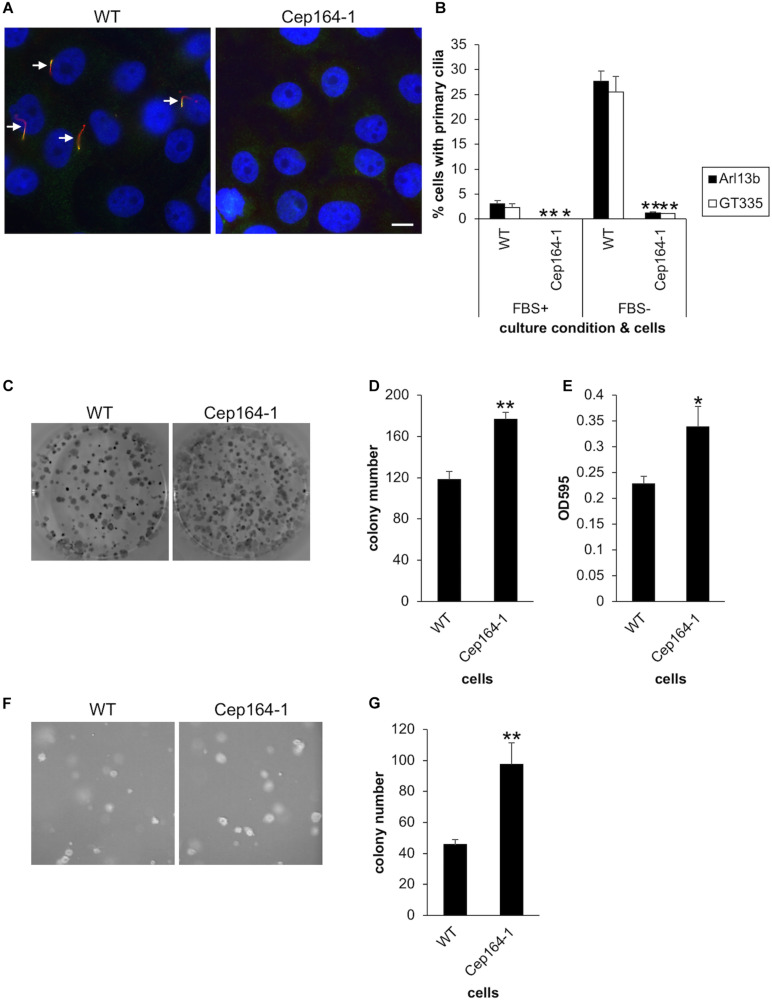
CEP164-mutated Panc1 cells show augmented colony-forming properties. **(A,B)** The indicated Panc1 cells were cultured in serum-fed (FBS+) or serum-starved medium (FBS–) for 48 h and immunostained with anti-glutamylated tubulin (GT335, green) and anti-Arl13b (red) antibodies. **(A)** Arrows indicate primary cilia. DNA was stained with Hoechst (blue). Scale bar, 10 μm. **(B)** The percentage of ciliated cells was determined. The average of three to four independent experiments is shown; >200 cells were scored each time. **(C–E)** The indicated cells were subjected to clonogenic assay. **(C)** Colonies were visualized with Crystal Violet and imaged. **(D)** The number of colonies was determined. The average of nine independent experiments is shown. **(E)** The OD at 595 nm of dissolved colonies was determined. The average of nine independent experiments is shown. **(F,G)** The indicated cells were subjected to soft agar assay. **(F)** Colonies were visualized with Crystal Violet and imaged. **(G)** The number of colonies was determined. The average of five independent experiments is shown. **(B,D,E,G)** All data are shown as mean ± SEM. **p < 0.01; *p < 0.05 compared with WT (two-tailed Student’s *t*-test).

To elucidate the biological effects of primary cilia loss in PDAC cells, we used Panc1 cells, originally isolated from primary tumors of PDAC (Lieber et al., 1975). Panc1 cells normally assemble primary cilia with low frequency, but their development is stimulated by serum starvation (Figures 1A,B; Nielsen et al., 2008). To obtain experimentally de-ciliated PDAC cells, we performed CRISPR/Cas9-mediated gene editing of CEP164 in Panc1 cells. Following this, sequencing analysis indicated three different alterations leading to premature nonsense codons in Cep164-1 cells (Supplementary Figure 1A). Nevertheless, immunofluorescence of CEP164 revealed faint CEP164 dots at centrioles (Supplementary Figure 1B), indicating that CEP164 was not completely knocked-out in this clone. The guide RNA (gRNA) against CEP164 targets a sequence in close proximity to the initial ATG codon (Supplementary Figure 1A). However, CEP164 lacking the N-terminal region could be translated from another ATG that resides downstream of a premature stop codon in Cep164-1 cells. Accordingly, we considered Cep164-1 as a CEP164 knock-down clone.

We then investigated whether our established Panc1 cells are able to assemble primary cilia. Immunofluorescence experiments with glutamylated tubulin (GT335) and ARL13B antibodies allowed visualization of primary cilia in Panc1 WT cells (Figures 1A,B). In contrast, Cep164-1 cells developed a few primary cilia, but the frequency was considerably lower than that of WT cells (Figures 1A,B). To investigate why Cep164-1 cells lose most of their primary cilia despite having measurable CEP164 at the centrosome, we performed immunostaining for tau tubulin kinase 2 (TTBK2), a kinase essential for ciliogenesis (Goetz et al., 2012). This revealed that TTBK2 was undetectable even at the basal body of primary cilia in Cep164-1 cells (Supplementary Figure 1C). Since TTBK2 is known to be recruited to the basal body through an interaction with the N-terminal region of CEP164, this result suggests that the N-terminal truncated CEP164 in Cep164-1 Panc1 cells is defective in tethering TTBK2 at the centriole, resulting in substantially reduced primary cilia.

To confirm that the observed de-ciliation in Cep164-1 cells was indeed due to depletion of CEP164, we subsequently performed rescue experiments. Primary cilia were significantly ameliorated by ectopic expression of CEP164 protein in Cep164-1 cells (Supplementary Figures 1B,D). These results indicate that the loss of function mutations in CEP164 lead to de-ciliation in Panc1 cells.

### Cep164-1 Panc1 Cells Show Enhanced Colony-Forming Ability *in vitro*

We next conducted an anchorage-dependent colony formation assay (clonogenic assay), which evaluates the reproductive viability of cells. This revealed that Cep164-1 cells formed more colonies than WT (Figures 1C,D), which was corroborated by the optical density (OD) of dissolved colonies (Figure 1E). Importantly, ectopic expression of CEP164 was sufficient to reverse the excess colony formation of Cep164-1 cells (Supplementary Figures 2A,B). These results suggest that CEP164 depletion induces enhanced clonogenicity of Panc1 cells. Next, we immunostained these colony-forming cells to evaluate primary cilia formation and the proportion of cycling cells. Approximately 20% of WT cells expressed primary cilia (Supplementary Figure 2C), indicating that despite the serum-fed cultivation conditions, ciliation is induced by the confluent state of the cells within colonies. The ratio of ciliated cells was inversely correlated with that of Ki67-positive cells in each WT colony (Supplementary Figure 2D). Contrastingly, cells in Cep164-1 colonies assembled a limited number of primary cilia, and coincidentally displayed higher Ki67 expression than WT (Supplementary Figure 2C). These data suggest that lack of primary cilia allows cell cycle progression in colony-forming Panc1 cells.

We subsequently performed an anchorage-independent colony formation assay (soft agar assay). Consistent with the previous clonogenic assay, we observed an increased number of colonies in Cep164-1 cells (Figures 1F,G). Ectopic CEP164 expression significantly overcame this phenotype in Cep164-1 cells (Supplementary Figure 2E). Collectively, these results suggest that ablation of CEP164 endows Panc1 cells with enhanced colony-forming ability.

### Chemical De-Ciliation Promotes Clonogenicity of Panc1 Cells

**FIGURE 2:**
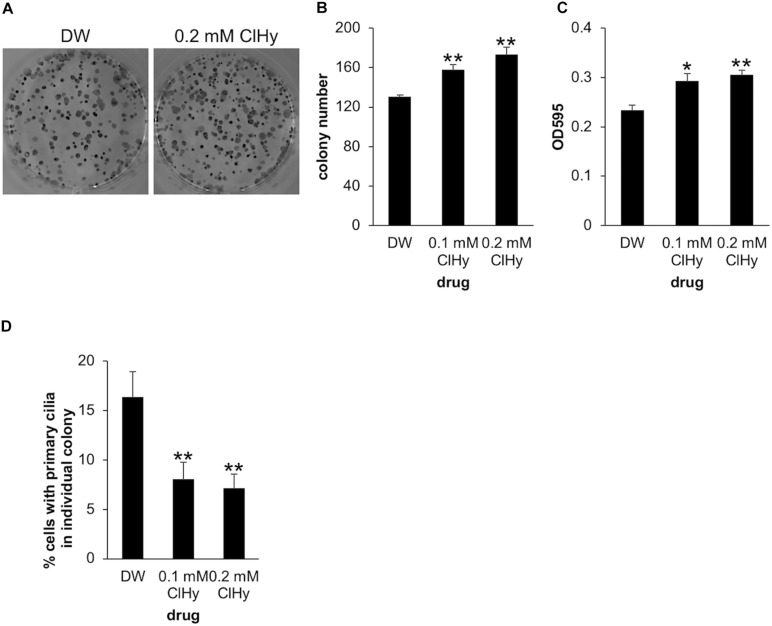
Chemical de-ciliation enhances clonogenicity of Panc1 cells. **(A–C)** Panc1 cells treated with the indicated concentration of chloral hydrate (ClHy) for 14 days were subjected to clonogenic assay. **(A)** Colonies were visualized with Crystal Violet and imaged. **(B)** The number of colonies was determined. The average of three to four independent experiments is shown. **(C)** The OD at 595 nm of dissolved colonies was determined. The average of three to four independent experiments is shown. **(D)** Colonies in the clonogenic assay were immunostained with an anti-Arl13b antibody. The percentages of cells with primary cilia in each colony was determined and their averages are shown. Number of colonies analyzed = 40 (DW), 45 (0.1 mM ClHy), 56 (0.2 mM ClHy). **(B–D)** All data are shown as mean ± SEM. **p < 0.01; *p < 0.05 compared with distilled water (DW) (two-tailed Student’s *t*-test).

To further examine the impact of de-ciliation on the clonogenic viability of Panc1 cells, we next investigated the effect of ClHy which is known to preclude primary cilia from the cell surface (Ho et al., 2013). Panc1 cells treated with ClHy formed a significantly high number of colonies in the clonogenic assay, phenocopying CEP164 depletion (Figures 2A,B). The ClHy-treated colonies showed higher OD than the control when dissolved in the 1% SDS solution (Figure 2C). We confirmed that ClHy reduces primary cilia in colony-forming cells as expected (Figure 2D). These results strongly suggest that de-ciliation potentiates the clonogenic competency of Panc1 cells.

### CEP164 Depletion Alters Cell Cycle Progression in Panc1 Cells

**FIGURE 3:**
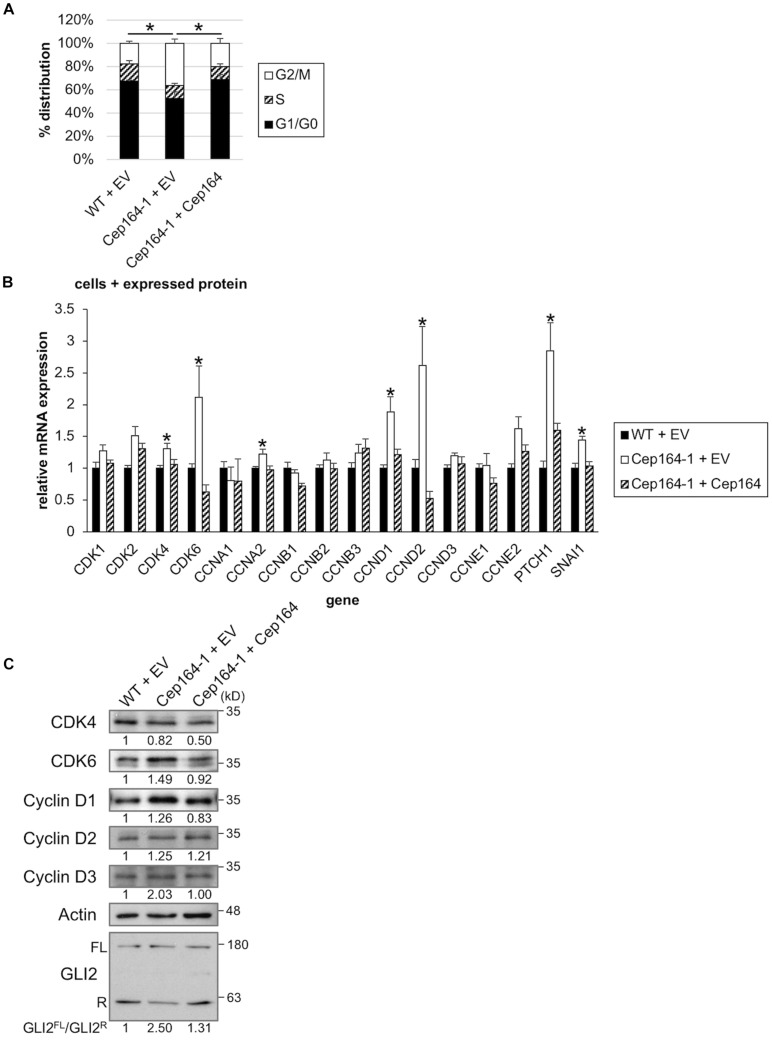
CEP164 depletion accelerates the cell cycle by elevation of Cyclin D-CDK6. **(A)** The indicated Panc1 cells were cultured in serum-fed medium for 48 h and analyzed using FACS. The proportion of cells at each cell cycle stage was determined. The average of three independent experiments is shown. **(B,C)** The indicated Panc1 cells were cultured in serum-fed medium for 48 h. **(B)** Relative amounts of the indicated mRNA were determined using quantitative PCR. GAPDH was used as a control. The average of four to eight independent experiments is shown. **(C)** Cell extracts were immunoblotted with indicated antibodies. β-Actin was used as a loading control. The amount of indicated proteins was quantified using ImageJ, and the relative values and GLI2^FL^/GLI2^R^ ratio are shown below the image. **(A,B)** All data are shown as mean ± SEM. *p < 0.05 compared with Cep164-1 + EV **(A)** (Chi-squared test), compared with WT + EV and Cep164-1 + Cep164 **(B)** (two-tailed Student’s *t*-test). Uncropped images of western blots are shown in Supplementary Figure 5.

We assessed the proliferative ability of Cep164-1 cells under normal culture condition. Although the percentages of nuclei positive for Ki67 were comparable between WT and Cep164-1 cells (Supplementary Figure 3A), FACS analysis revealed an increase in G2/M phase and a concurrent decrease in G0/G1 phase in Cep164-1 cells (Figure 3A), suggesting a high proliferative potential compared to WT cells. Given that Panc1 cells assemble less than 4% of primary cilia in serum-fed cultivation (Figure 1B), this cell cycle alteration is likely to occur independent of de-ciliation in Cep164-1 cells. No impact on cell cycle of ClHy-treated Panc1 cells, irrespective of ciliation decrease, was observed to support this hypothesis (Supplementary Figures 3B,C). To investigate possible explanations for Cep164-1 cell cycle change, we evaluated the expression of major CDKs and Cyclins using quantitative PCR. We detected significant elevations in cyclin dependent kinase 4 (CDK4), CDK6, Cyclin A2/CCNA2, Cyclin D1/CCND1, and Cyclin D2/CCND2 expression in Cep164-1 (Cep164-1 + EV) cells compared to that in WT (WT + EV) and rescue (Cep164-1 + Cep164) cells (Figure 3B). In particular, CDK6, Cyclin D1, and Cyclin D2 were substantially increased in Cep164-1 cells. We further detected augmented protein expression of Cyclin D1, Cyclin D3, and CDK6 in Cep164-1 cells by immunoblot analysis (Figure 3C). In contrast, ClHy-treatment did not enhance Cyclin D-CDK4/6 expression (Supplementary Figure 3D). These data suggest that CEP164 depletion hastens cell cycle progression through excess expression of D-type Cyclins-CDK6, probably independent of de-ciliation.

### Ablation of CEP164 Induces Activation of GLI2 Transcription Factor of the Hh Pathway

**FIGURE 4:**
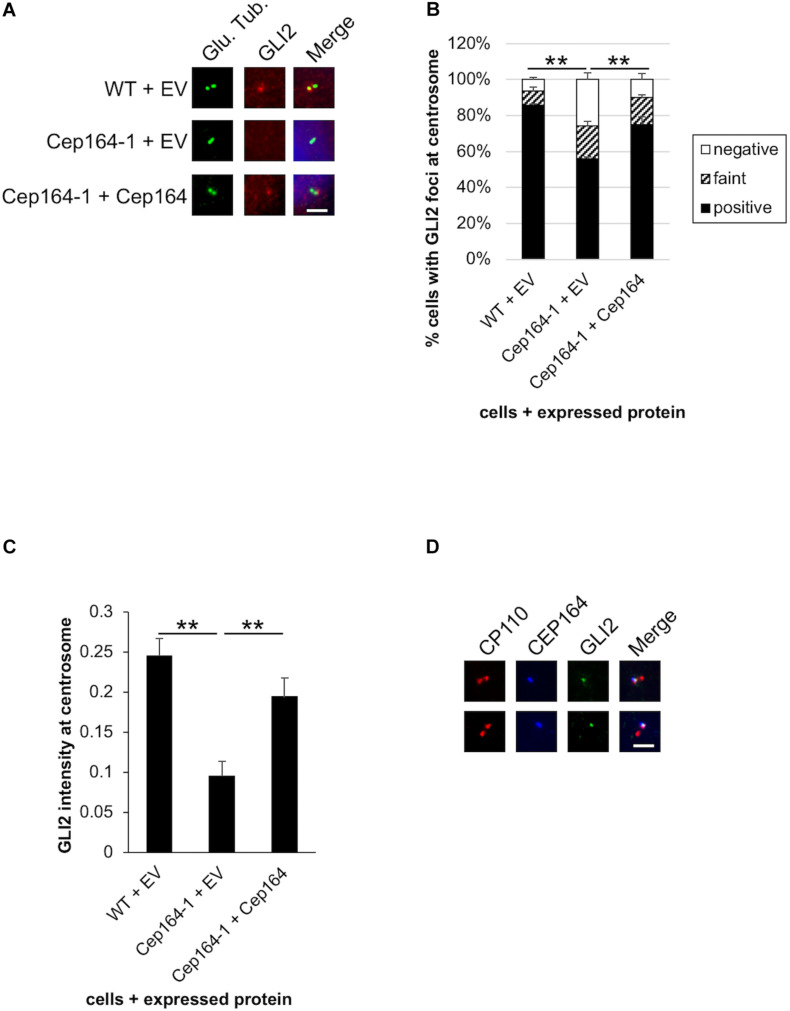
GLI2 dot disappears from the mother centriole in CEP164-mutated Panc1 cells. **(A–C)** The indicated cells were cultured in serum-fed medium for 48 h and immunostained with anti-glutamylated tubulin (green) and anti-GLI2 (red) antibodies. **(A)** DNA was stained with Hoechst (blue). Scale bar, 2.5 μm. **(B)** The percentage of cells with GLI2 dot at the non-ciliated centrosome was determined. The average of three independent experiments is shown; >100 cells were scored each time. **(C)** The quantified fluorescence intensity of GLI2 at centrosome is shown. *n* = 31 (WT + EV), 25 (Cep164-1 + EV), 35 (Cep164-1 + Cep164). **(D)** Panc1 cells were cultured in serum-fed medium for 48 h and immunostained with anti-CP110 (red), anti-CEP164 (blue), and anti-GLI2 (green) antibodies. Two representative images are shown. Scale bar, 2.5 μm. **(B,C)** All data are shown as mean ± SEM. **p < 0.01 compared with Cep164-1 + EV **(B)** (Chi-squared test), compared with Cep164-1 + EV **(C)** (two-tailed Student’s *t*-test).

CCND1, CCND2, and CDK6 are target genes of Hh signaling (Katoh and Katoh, 2009; Raleigh et al., 2018). We also validated mRNA expression of known Hh-related genes, Patched 1/PTCH1 and snail family transcription repressor 1 (Snail/SNAI1), and found them to be up-regulated in Cep164-1 cells (Figure 3B). These data suggest that the Hh pathway is activated in Cep164-1 cells. As GLI2 is the principal regulator among GLI family of transcription factors which mediate the Hh signaling (Briscoe and Thérond, 2013), we investigated GLI2 expression and detected GLI2 dot at non-ciliated centriole in Panc1 cells using immunofluorescence analysis (Figure 4A). This GLI2 dot was significantly diminished in Cep164-1 cells (Figures 4A–C), suggesting that CEP164 is required for GLI2 to locate at centrioles without primary cilia. Importantly, GLI2 overlapped with CEP164 at the mother centriole (Figure 4D). Furthermore, immunoblot analysis using a previously validated GLI2 antibody that recognizes both full length and repressor form of GLI2 (GLI2^FL^ and GLI2^R^, respectively) showed that the amount of GLI2^R^ was reduced, thereby causing GLI2 activation (increase in GLI2^FL^/GLI2^R^) in Cep164-1 cells (Figure 3C; Nielsen et al., 2008). Altogether, these data suggest that CEP164 depletion abrogates GLI2 localization to the mother centriole and production of GLI2^R^, inducing GLI2 activation and Cyclin D-CDK6 expression.

### Cep164-1 Cells Are Tolerant to Growth Retardation Provoked by KRAS Depletion

**FIGURE 5:**
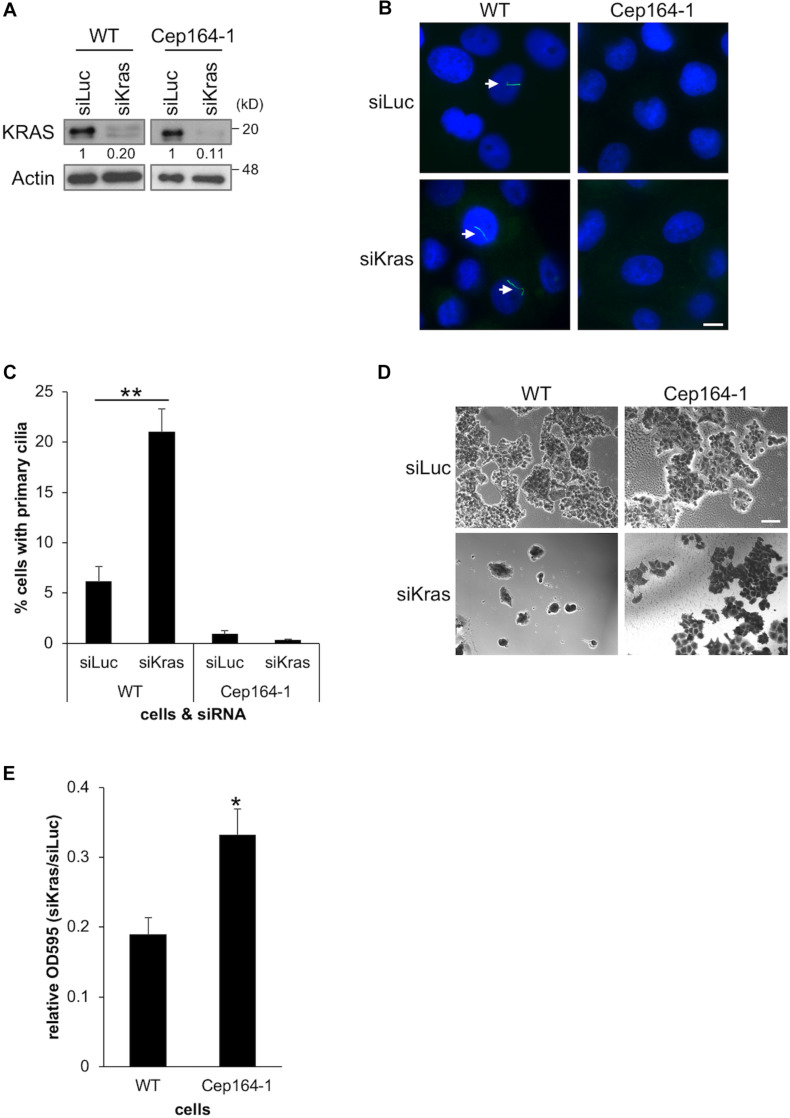
Cep164-1 cells are moderately tolerant to growth delay provoked by KRAS depletion. **(A–E)** The indicated cells transiently transfected with siLucifearase (Luc) or siKras were cultured for 5 days. **(A)** Cell extracts were immunoblotted with an anti-KRAS antibody. β-Actin was used as a loading control. The amount of indicated proteins was quantified using ImageJ, and the relative values are shown below the image. **(B)** Cells were immunostained with an anti-Arl13b (green) antibody. DNA was stained with Hoechst (blue). White arrows indicate primary cilia. Scale bar, 10 μm. **(C)** The percentage of ciliated cells was determined. The average of three independent experiments is shown; >200 cells were scored each time. **(D)** Cells were subjected to Crystal Violet assay. Representative images are shown. Scale bar, 100 μm. **(E)** The OD at 595 nm of dissolved cells was determined. The average of five independent experiments is shown. **(C,E)** All data are shown as mean ± SEM. **p < 0.01; *p < 0.05 compared with siLuc **(C)** or WT **(E)** (two-tailed Student’s *t*-test). Uncropped images of western blots are shown in Supplementary Figure 5.

Constitutively active KRAS is a hallmark of PDAC which is expressed in Panc1 cells. Several studies have demonstrated that acute KRAS silencing hampers the proliferation of PDAC cell lines (Brummelkamp et al., 2002; Singh et al., 2009; Collisson et al., 2011). We previously reported that siRNA-mediated silencing of KRAS restores primary cilia and thereby disrupts Ki67 expression in Panc1 cells (Kobayashi et al., 2017). Based on these studies, we investigated whether KRAS depletion suppresses the growth of Cep164-1 cells which have limited ability of ciliogenesis, and conducted siRNA-mediated silencing of KRAS in Cep164-1 cells (Figure 5A). Knock-down of KRAS restored primary cilia formation and concurrently impeded the proliferation of Panc1 cells (Figures 5B–E). In contrast, KRAS ablation did not restore primary cilia in Cep164-1 cells and had lesser effect on their proliferation (Figures 5B–E). Ectopic CEP164 expression significantly reversed this phenotype in Cep164-1 cells (Supplementary Figure 4). These results suggest that the inhibitory effect of KRAS ablation on the proliferation of PDAC cells partly depends on CEP164 expression.

## Discussion

Primary cilia in Panc1 cells contain signaling proteins such as PTCH1 and smoothened (SMO) which suggests that they maintain their functional integrity (Nielsen et al., 2008). We targeted CEP164 to obtain de-ciliated Panc1 cells since this protein is known to be required for ciliogenesis in mammalian cells (Graser et al., 2007; Schmidt et al., 2012; Daly et al., 2016; Siller et al., 2017). Chemical elimination of primary cilia as well as CEP164 depletion significantly enhanced the clonogenicity of Panc1 cells, allowing us to conclude that over-proliferation, at least in part, occurs as a result of de-ciliation in PDAC cells. Based on our data and the results of a previous study (Deng et al., 2018), primary cilia appear to be suppressors of PDAC proliferation. This notion is supported by the fact that PDAC cells gradually lose primary cilia over time (Schimmack et al., 2016; Deng et al., 2018). Conversely, a clinical report showed that the presence of primary cilia correlates with poor prognosis of PDAC patients (Emoto et al., 2014), suggesting the possibility of a promotive role of primary cilia. Further studies will be needed to clarify the circumstances under which the primary cilia promote or suppress PDAC growth *in vivo* as well as *in vitro*.

Thus far, several reports have shown cell cycle variation in cells depleted of CEP164 (Sivasubramaniam et al., 2008; Chaki et al., 2012; Slaats et al., 2014; Liu et al., 2017); however, the distribution of each phase is inconsistent among cell types and the molecular mechanism remains unknown. Herein, we discovered a notable increase in Cyclin D-CDK6 as well as cell cycle alteration in CEP164-mutated Panc1 cells. These phenotypes are observed in serum-fed cultivation in which primary cilia are rarely assembled in Panc1 cells. In addition, ClHy-dependent reduction of primary cilia did not impact the cell cycle and Cyclin D-CDK6 expression. These results strongly indicate that CEP164 contributes to the cell cycle regulation independent of primary cilia. Given that Cyclin D1 and Cyclin D3 expression is much higher than that of Cyclin D2 in PDAC cells (Radulovich et al., 2010), these D-type Cyclins appear to play a predominant role in PDAC cell proliferation. In line with Cyclin D-CDK6 complex regulation of the G1-S transition of the cell cycle, the distribution of the G0/G1 phase is decreased in Cep164-1 cells. A previous study has demonstrated that CDK6 is a transcriptional target of GLI2 (Raleigh et al., 2018). Cyclin D1 and Cyclin D2 are also known to be target genes of the Hh pathway (Katoh and Katoh, 2009). Based on the available knowledges and our data, we focused on GLI2. While GLI2 is known to localize to the ciliary shaft and tip of primary cilia in PDAC cells (Nielsen et al., 2008), its localization in non-ciliated cells remains to be identified. We detected GLI2 foci almost overlapping with CEP164 at the DA of the mother centriole without primary cilia. Moreover, we found that CEP164 depletion induces reduction of GLI2 from the mother centriole and GLI2 activation. These findings suggest a mechanistic model in which CEP164 tethers and/or recruits GLI2 at the DA of the mother centriole to produce GLI2^R^. Protein kinase A (PKA) regulates phosphorylation and activation of GLI2 at the basal body of primary cilia in mouse embryo (Tuson et al., 2011). Besides, DAZ-interacting zinc finger protein 1 (DZIP1) has been reported to be located at the basal body, and is required for CEP164 localization and GLI2 activation in mouse embryo (Wang et al., 2013). Therefore, combined with these proteins, CEP164 might control GLI2 truncation irrespective of presence or absence of primary cilia in PDAC cells.

The present study suggests that decreased CEP164 expression is favorable for PDAC proliferation through a dual mechanism, namely de-ciliation and primary cilium-independent GLI2-Cyclin D/CDK6 activation. The fact that low CEP164 expression is a poor prognostic factor in pancreatic cancer patients supports this hypothesis (Uhlen et al., 2017). Our data suggest that PDAC cells that lack CEP164 are less sensitive to growth inhibition provoked by KRAS silencing. Although KRAS and its downstream effectors are promising therapeutic targets of PDAC, there are still no effective approaches (Waters and Der, 2018). Our findings offer the potential to improve PDAC treatment. Since CEP164 loss induces excess Cyclin D-CDK6 expression in PDAC cells, concurrent treatment of CDK6 inhibitors with KRAS-targeting therapy could be worth pursuing. Besides, considering the de-ciliation-dependent over-proliferation of CEP164-depleted PDAC cells, KRAS-targeted therapy might be effective in combination with an agent which ameliorates ciliogenesis. HDAC inhibitors are known to restore primary cilia in PDAC cells (Kobayashi et al., 2017), and combinatorial inhibition of KRAS-effectors and HDACs inhibits PDAC growth considerably more than singular treatment (Ischenko et al., 2015). It may also be possible to utilize ciliogenic compounds effective for several cancer cells in a similar manner (Khan et al., 2016). Further studies aimed at defining the relevance of ciliation or de-ciliation to the efficacy of various anticancer treatments may lead to better strategies to treat the intractable cancer, PDAC.

Further investigations are warranted to determine how de-ciliation induces PDAC cells proliferation. Loss of primary cilia has been shown to potentiate the MVA pathway thereby promoting the transformation of pancreatic ductal cells (Deng et al., 2018); however, we were unable to detect genes up-regulation of the pathway in the Cep164-1 and ClHy-treated cells (data not shown). Unidentified centrioles- and/or primary cilia-dependent signaling might be involved in this process. Alternatively, detached centrioles from plasma membrane could accelerate the cell cycle, especially during mitosis, which is a subject for future studies.

## Materials and Methods

### Cell Culture

Panc1 (American Type Culture Collection), Cep164-1 Panc1 (this study), and Lenti-X 293T (gift from M. Hagiwara) cells were grown in Dulbecco’s Modified Eagle Medium (DMEM) (Nacalai Tesque) supplemented with 10% Fetal Bovine Serum (FBS) (Biosera) and 100 units/ml penicillin and 100 μg/ml streptomycin (P/S) (Nacalai Tesque). Panc1 cells were established from primary tumors of PDAC and are known to assemble primary cilia (Lieber et al., 1975; Nielsen et al., 2008).

### Antibodies and Reagents

Antibodies used in this study include mouse anti-glutamylated tubulin (GT335) (1:1000, Adipogen, AG-20B-0020), rabbit anti-ARL13B (1:1000, Proteintech, 17711-1-AP), mouse anti-ARL13B (1:1000, NeuroMab, 75-287), mouse anti-CEP164 (1:500, Santa Cruz, sc-515403), rabbit anti-Ki67 (1:2000, Abcam, ab15580), rabbit anti-TTBK2 (1:500, Sigma-Aldrich, HPA018113), rabbit anti-CP110 (1:1000, gift from B. D. Dynlacht), rabbit anti-KRAS (1:2000, Proteintech, 12063-1-AP), rabbit anti-CDK4 (1:400, Santa Cruz, sc-260), mouse anti-CDK6 (1:400, Santa Cruz, sc-7961), mouse anti-Cyclin D1 (1:400, Santa Cruz, sc-8396), mouse anti-Cyclin D2 (1:400, Santa Cruz, sc-53637), mouse anti-Cyclin D3 (1:400, Santa Cruz, sc-6283), goat anti-GLI2 [1:100 (IF), 1:400 (WB), Santa Cruz, sc-271786], and mouse anti-β-Actin (1:1000, Santa Cruz, sc-47778). Reagents used in this study include Chloral Hydrate (Nacalai Tesque, 07922-62), Hoechst 33342 (Nacalai Tesque, 04915-82), and Propidium Iodide (PI) (Nacalai Tesque, 29037-76).

### Plasmids

To generate gRNA targeting CEP164, annealed oligo was inserted into PX458 [pSpCas9(BB)-2A-GFP] (Addgene) (Ran et al., 2013). Oligo DNAs are listed in Supplementary Table 1.

To generate Flag-CEP164, human CEP164 fragment encoding residue 1-4383 was excised from pEGFP-N3-CEP164 (gift from S. Chiba) and sub-cloned into pLVX-3Flag-IRES-Puro (gift from B. D. Dynlacht).

Plasmid transfection into Panc1 and Lenti-X 293T cells was performed using Lipofectamine 2000 (Invitrogen) and PEI Max (Polysciences) according to the manufacturer’s instruction, respectively.

### Generation of Cep164-1 Cells

PX458-CEP164 plasmid was transfected into Panc1 cells using Lipofectamine 2000. Transfected cells were cultured for 72 h and singly sorted into 96-well plates by GFP signal using FACSAria (BD biosciences). Genome DNA was extracted from survival cells using QuickExtract DNA Solution 1.0 (epicentre), and amplified PCR products using primers listed in Supplementary Table 1 were sub-cloned into pGEM-T Easy (Promega). Purified plasmid DNAs were sequenced using M13 primers.

### Generation of Rescue Cells

Lentivirus supernatant was produced by co-transfection of pLVX-IRES-Puro [Empty Vector (EV)] or pLVX-3Flag-CEP164-IRES-Puro (CEP164) with Δ8.9, pcRev, and VSVG plasmids (gift from M. Hagiwara) into Lenti-X 293T cells using PEI Max. The virus supernatant was harvested 72 h post-transfection and concentrated using Lenti-X Concentrator (Clonetech). Panc1 cells were incubated with virus in the presence of 5 μg/ml polybrene (Nacalai Tesque) for 72 h. The infected Panc1 cells were subsequently cultured in medium with 5 μg/ml puromycin (Nacalai Tesque) for 8 days. Established cells (WT + EV, Cep164-1 + EV, and Cep164-1 + Cep164) were cultured in medium with 3 μg/ml puromycin.

### Proliferation Assay

For crystal violet assay [growth assay on 2-dimensional (2D) plastic dish, Figure 5), 2 × 10^3^ cells were seeded in 96-well plate and cultured for 24 h. Following siRNA transfection, cells were cultured for 5 days. Cells were washed with phosphate-buffered saline (PBS) twice and stained with 0.2% Crystal Violet (Nacalai Tesque) in 20% MeOH for 10 min. After washing with PBS four times, cells were observed and imaged using AxioObserver (Zeiss) with a 10× lens. Cells were subsequently dissolved with 1% SDS and OD was measured at 595 nm.

For clonogenic assay (colony formation on 2D plastic dish), 5 × 10^2^ cells were seeded in 12-well plate and cultured for 14 days. Staining with Crystal Violet and following measurement of OD were performed as described in crystal violet assay. Number of colonies was determined using phase contrast microscope and photographed using LAS4000 (FujiFilm).

For soft agar colony formation assay, 1 × 10^3^ cells were mixed with 0.7% Agar/medium on 1% Agar/medium in 6-well plate. A 1-ml medium was overlaid on the gel to avoid drying. After 14 days, colonies were stained with 0.02% Crystal Violet in 20% MeOH for 2 h. Number of colonies was determined using phase contrast microscope.

### RNAi

Synthetic siRNA oligonucleotides were obtained from Dharmacon. The siRNA for luciferase (siLuc) was 5′-CGTACGCGGAATACTTCGA-3′, while that for Kras was 5′-GGAGGGCTTTCTTTGTGTA-3′ (Kobayashi et al., 2017). Three picomoles siRNA was transfected into Panc1 cells in 96-well plate using Lipofectamine RNAiMAX (Invitrogen) according to the manufacturer’s instruction.

### Western Blotting

Cells were lysed with lysis buffer (50 mM Hepes pH 7.5, 150 mM NaCl, 5 mM EDTA, 0.5% NP-40, 10% Glycerol, 1 mM DTT, 0.5 mM PMSF, 2 μg/ml leupeptin, 5 mM NaF, 10 mM β-Glycerophosphate, and 1 mM Na_3_VO_4_) at 4°C for 30 min. A 20 μg (Figure 3C) or 6 μg (Figure 5A) lysate was loaded and analyzed using SDS-PAGE and immunoblotting. Pixel intensities of the images were quantified using ImageJ.

### Immunofluorescence Microscopy

Immunofluorescence microscopy was conducted as previously described (Dateyama et al., 2019). Briefly, cells were fixed with cold methanol for 5 min or 4% PFA in PBS for 10 min, and permeabilized with 0.2% Triton X-100/PBS for 10 min. Slides were blocked with 5% BSA/PBS prior to incubation with primary antibodies. Primary and secondary antibodies were diluted to the desire concentrations using 5% BSA/PBS. Secondary antibodies used were AlexaFluor350-, AlexaFluor488-, or AlexaFluor594- conjugated donkey anti-mouse, anti-rabbit, or anti-goat IgG (Invitrogen). Cells were stained with Hoechst33342 to visualize DNA. Mounted slides with PermaFluor Mounting Medium (Thermo Fisher Scientific) were observed and imaged using AxioObserver with a 63× lens. The pixel intensities of GLI2 in proximity to centrosome were quantified using ImageJ as previously described (Kobayashi et al., 2014).

### Flow Cytometry

Cells were fixed with cold ethanol for 30 min, stained with PI for 45 min, and analyzed using FACS Calibur (BD Biosciences). Data were analyzed using FlowJo software (FlowJo).

### Quantitative PCR

Total RNA was isolated from cultured cells using Sepasol (Nacalai Tesque), and following reverse transcription reaction was performed using ReverTra Ace qPCR RT kit (TOYOBO). Quantitative PCR (qPCR) was performed using THUNDERBIRD SYBR qPCR mix (TOYOBO) and LightCycler96 (Roche). All reaction was conducted according to the manufacturer’s instruction. Primers are listed in Supplementary Table 2.

### Statistical Analysis

The statistical significance of the difference was determined using two-tailed Student’s *t*-test (except for Figures 3A, 4B and Supplementary Figure 3C) or Chi-squared test (Figures 3A, 4B and Supplementary Figure 3C). Figure legends indicate the number of independent replicates conducted and the number of cells analyzed for each replicate. Differences were considered significant when *p* < 0.05. ^∗∗^*p* < 0.01; ^∗^*p* < 0.05.

## Data Availability Statement

The raw data supporting the conclusions of this article will be made available by the authors, without undue reservation.

## Author Contributions

TK, KT, YM, AS, and MT performed the experiments. TK coordinated the study and oversaw all experiments. TK and HI wrote the manuscript. All authors discussed the results, commented on the manuscript, contributed to the article, and approved the submitted version.

## Conflict of Interest

The authors declare that the research was conducted in the absence of any commercial or financial relationships that could be construed as a potential conflict of interest.
