# Supplementary material for: CEP164 Deficiency Causes Hyperproliferation of Pancreatic Cancer Cells
Source: Front Cell Dev Biol. 2020 Nov 5;8:587691. doi: 10.3389/fcell.2020.587691 (PMC7674857; doi:10.3389/fcell.2020.587691)
Supplement: Supplementary file 1 [file Data_Sheet_1.docx]

**Supplemental Figure legends**

**Figure S1**

**Mutations in Cep164-1 Panc1 cells**

**(A)** (left) Three different mutations (alleles A, B, and C) of the CEP164 gene in Cep164-1 Panc1 cells (note that Panc1 cells are frequently triploid). Red sequences represent the target of guide RNAs with underlined protospacer adjacent motif (PAM). Bold ATG is the start codon of CEP164 cDNA. Blue sequences show insertions at X in alleles B and C. Asterisks under italicized sequences indicate premature stop codons. (right) Proteins translated from each clone. Green shows incorrect residues. **(B, C)** The indicated Panc1 cells were immunostained with (B) anti-CP110 (red) and anti-CEP164 (green), or (C) anti-glutamylated tubulin (green) and anti-TTBK2 (red) antibodies. DNA was stained with Hoechst (blue). Scale bars, 5 µm. **(D)** The indicated Panc1 cells were cultured in serum-starved medium for 48 h and immunostained with an anti-Arl13b antibody. The percentage of ciliated cells was determined. The average of three to five independent experiments is shown; >200 cells were scored each time.

**(D)** All data are shown as mean ± SEM. **, *p* < 0.01 compared with Cep164-1 + EV (two-tailed Student’s *t*-test).

**Figure S2**

**Ectopic CEP164 expression rescues over-clonogenicity of Cep164-1 cells**

**(A, B)** The indicated Panc1 cells were subjected to clonogenic assay. (A) Colonies were visualized with Crystal Violet and imaged. The average of four independent experiments is shown. (B) The OD at 595 nm of dissolved colonies was determined. The average of four independent experiments is shown. **(C, D)** Colonies in the clonogenic assay were immunostained with anti-Arl13b and anti-Ki67 antibodies. (C) The percentages of cilia- or Ki67-positive cells in each colony were determined and their averages are shown. Number of colonies analyzed = 36 (WT), 30 (Cep164-1). (D) The percentages of cilia- or Ki67-positive cells in each WT colony are individually plotted. r, Pearson correlation coefficient. **(E)** The indicated Panc1 cells were subjected to soft agar assay. Colonies were visualized with Crystal Violet and imaged, then the number of colonies was determined. The average of four independent experiments is shown.

**(A-C, E)** All data are shown as mean ± SEM. **, *p* < 0.01; *, *p* < 0.05 compared with Cep164-1 + EV (A, B, E) or WT (C) (two-tailed Student’s *t*-test).

**Figure S3**

**ClHy-dependent de-ciliation does not impinge on cell cycle of Panc1 cells**

**(A)** The indicated Panc1 cells were cultured for 48 h and then immunostained with an anti-Ki67 antibody. The percentage of cells with Ki67-positive nuclei was determined. The average of three independent experiments is shown; >200 cells were scored each time. **(B-D)** Panc1 cells were treated with the indicated concentration of ClHy for 48 hrs. (B) Cells were immunostained with an anti-Arl13b antibody. The percentage of ciliated cells was determined. The average of three independent experiments is shown; >200 cells were scored each time. (C) The proportion of cells at each cell cycle stage was determined using FACS. The average of three independent experiments is shown. (D) Relative amounts of the indicated mRNA were determined using quantitative PCR. GAPDH was used as a control. The average of three independent experiments is shown.

**(A-D)** All data are shown as mean ± SEM. *, *p* < 0.05 compared with DW (B) (two-tailed Student’s *t*-test). NS, no significance.

**Figure S4**

**Ectopic CEP164 expression reverses tolerance to KRAS depletion in Cep164-1 cells**

The indicated cells transiently transfected with siLucifearase (Luc) or siKras were cultured for 5 days and subjected to Crystal Violet assay. The optimal density at 595 nm of dissolved cells was determined. The average of four independent experiments is shown.

All data are shown as mean ± SEM. *, *p* < 0.05 compared with Cep164-1 + EV (two-tailed Student’s *t*-test).

**Figure S5**

**Uncropped images of western blots**
