# Supplementary material for: CEP164 Deficiency Causes Hyperproliferation of Pancreatic Cancer Cells
Source: Front Cell Dev Biol. 2020 Nov 5;8:587691. doi: 10.3389/fcell.2020.587691 (PMC7674857; doi:10.3389/fcell.2020.587691)
Supplement: Supplementary file 2 [file Table_1.DOCX]

| **Name** | **Sequence (5' to 3')** | **use** |
| --- | --- | --- |
| **hCep164KO_F** | **CACCGCCAGCTGATCTCCTATGCGG** | **guide RNA** |
| **hCep164KO_R** | **AAACCCGCATAGGAGATCAGCTGGC** | **guide RNA** |
| **hCep164check_F** | **GAGTTGTTGGTTTCACCCTC** | **Genome PCR** |
| **hCep164check_R** | **GCCAAGGTAGGCTCACATTT** | **Genome PCR** |

**Table S1**

**Primers used for generation of Cep164-1 cells**
