# Supplementary material for: CEP164 Deficiency Causes Hyperproliferation of Pancreatic Cancer Cells
Source: Front Cell Dev Biol. 2020 Nov 5;8:587691. doi: 10.3389/fcell.2020.587691 (PMC7674857; doi:10.3389/fcell.2020.587691)
Supplement: Supplementary file 3 [file Table_2.DOCX]

| **Name** | **Sequence (5' to 3')** |
| --- | --- |
| **hCDK1_F** | **AGAAAGTGAAGAGGAAGGGGT** |
| **hCDK1_R** | **GAGATATAACCTGGAATCCTGCA** |
| **hCDK2_F** | **TCACTGGCATTCCTCTTCCC** |
| **hCDK2_R** | **ACCCGATGAGAATGGCAGAA** |
| **hCDK4_F** | **TCCCGAAGTTCTTCTGCAGT** |
| **hCDK4_R** | **GTCGGCTTCAGAGTTTCCAC** |
| **hCDK6_F** | **CGAACAGACAGAGAAACCAAAC** |
| **hCDK6_R** | **AAGAAAGTCCAGACCTCGGA** |
| **hCCNA1_F** | **AATGGGCAGTACAGGAGGAC** |
| **hCCNA1_R** | **GCTGGAGGGAAGGCATTTTC** |
| **hCCNA2_F** | **GGTACTGAAGTCCGGGAAC** |
| **hCCNA2_R** | **GGAACGGTGACATGCTCATC** |
| **hCCNB1_F** | **ACCTGAGCCTGTTAAAGAAGAA** |
| **hCCNB1_R** | **CAGGTCTTCTTCTGCAGGGG** |
| **hCCNB2_F** | **ACTCTGTACATGTGCGTTGG** |
| **hCCNB2_R** | **AGAGCAGAGCAGTAATCCCA** |
| **hCCNB3_F** | **TGAGACCCTGTACTTGGCAG** |
| **hCCNB3_R** | **CAAAGTCATCCACACGAGGT** |
| **hCCND1_F** | **ACAGATCATCCGCAAACACG** |
| **hCCND1_R** | **ATGGAGGGCGGATTGGAAAT** |
| **hCCND2_F** | **GCGGAGAAGCTGTGCATTTA** |
| **hCCND2_R** | **CACTTCAACTTCCCCAGCAC** |
| **hCCND3_F** | **TTGCACATGATTTCCTGGCC** |
| **hCCND3_R** | **ATCATGGATGGCGGGTACAT** |
| **hCCNE1_F** | **ATGGCCAAAATCGACAGGAC** |
| **hCCNE1_R** | **TTTGTCAGGTGTGGGGATCA** |
| **hCCNE2_F** | **TCCTTCACCTTTGCCTGATT** |
| **hCCNE2_R** | **CCTCATCTGTGGTTCCAAGTC** |
| **hPTCH1_F** | **AGCGGTAGTAGTGGTGTTCA** |
| **hPTCH1_R** | **CTGAATCACTCTGCTGACGC** |
| **hSNAI1_F** | **CATGTCCGGACCCACACT** |
| **hSNAI1_R** | **CATCTGAGTGGGTCTGGAGG** |
| **hGAPDH_F** | **GGCTGAGAACGGGAAGCTTG** |
| **hGAPDH_R** | **ACTCCACGACGTACTCAGCG** |

**Table S2**

**Primers used for quantitative PCR**
