# Supplementary figures and images for: CEP164 Deficiency Causes Hyperproliferation of Pancreatic Cancer Cells
Source: Front Cell Dev Biol. 2020 Nov 5;8:587691. doi: 10.3389/fcell.2020.587691 (PMC7674857; doi:10.3389/fcell.2020.587691)

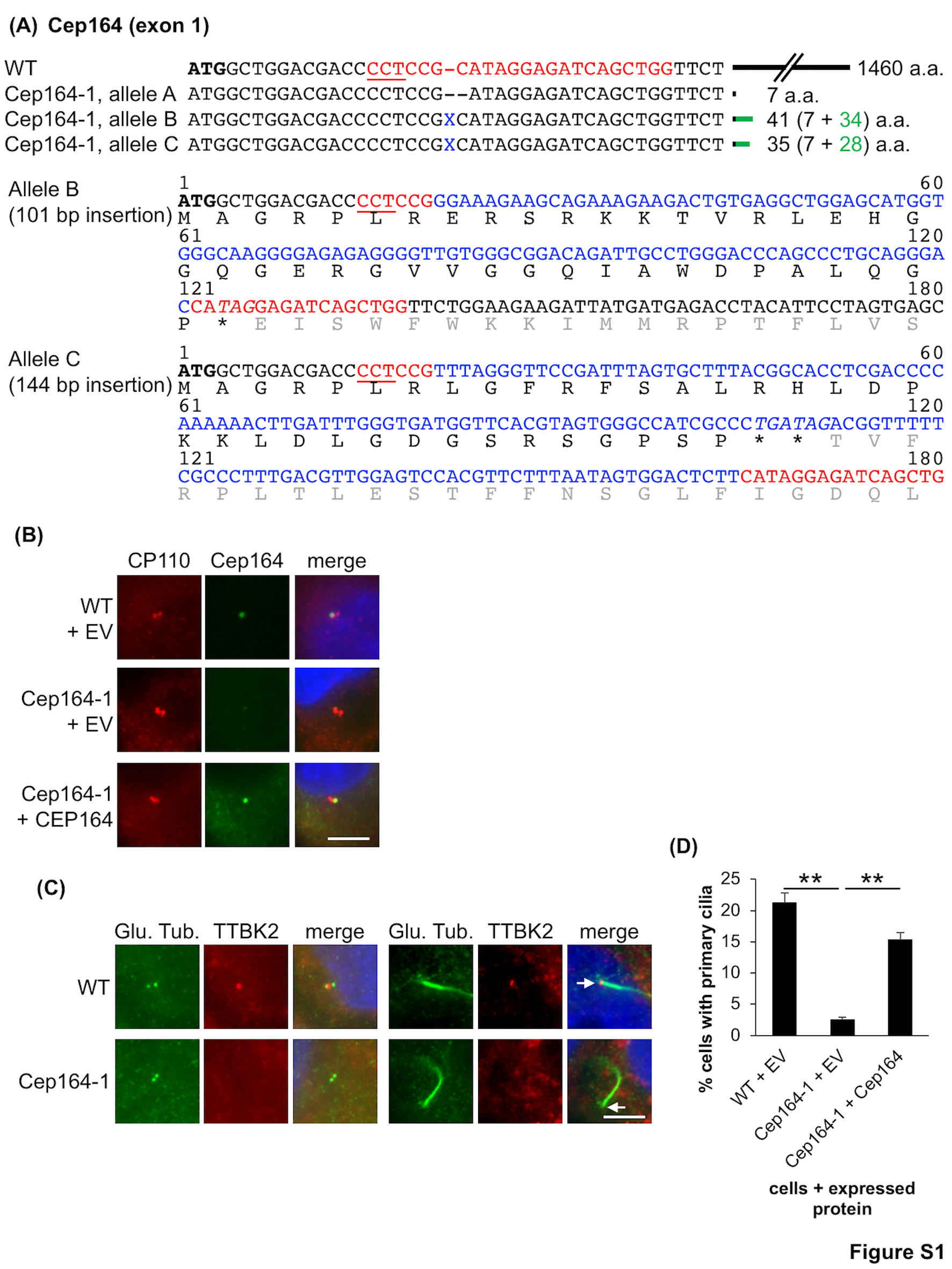

Supplement: Supplementary file 5 [file Image_1.TIFF]

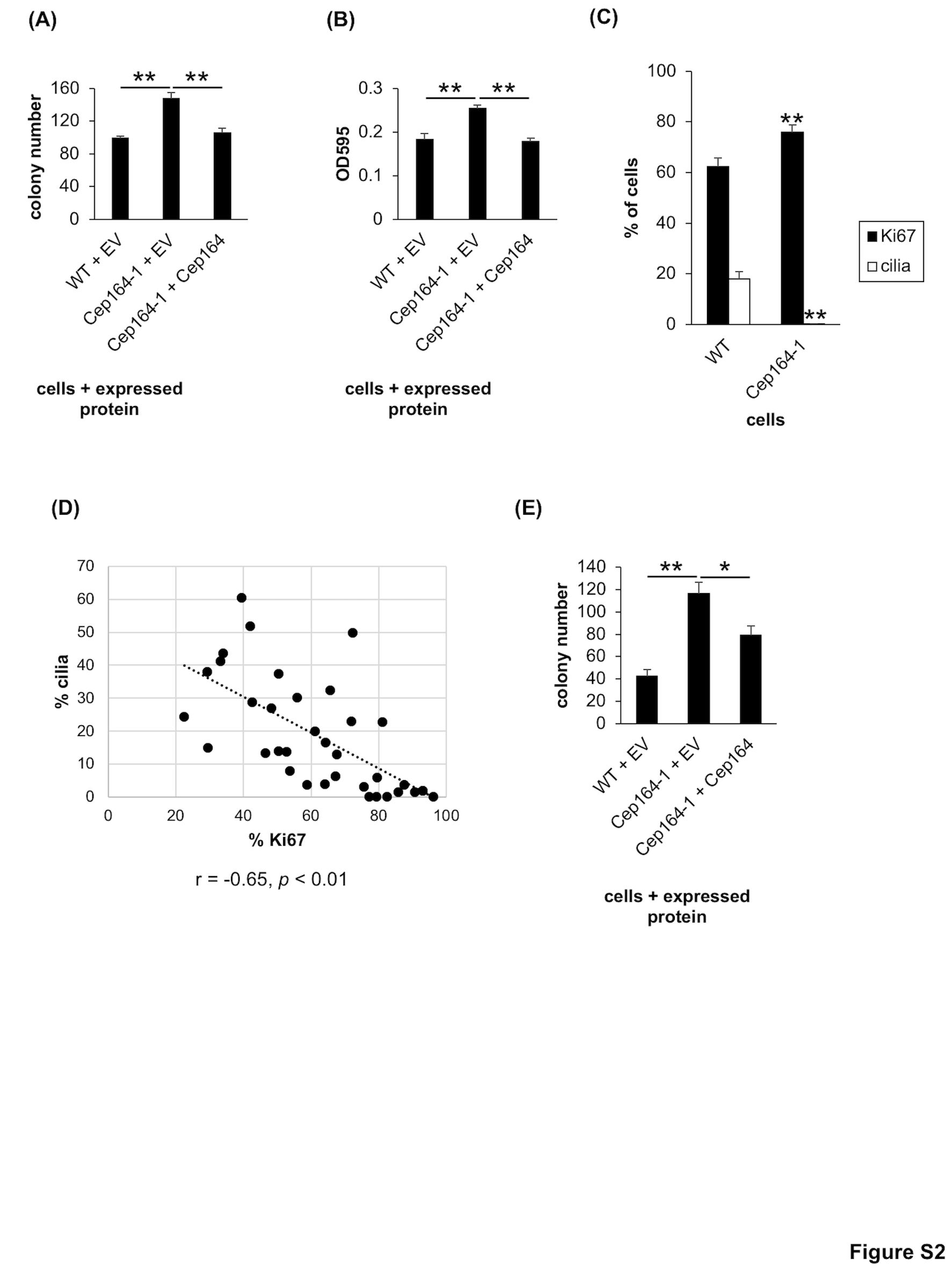

Supplement: Supplementary file 6 [file Image_2.TIFF]

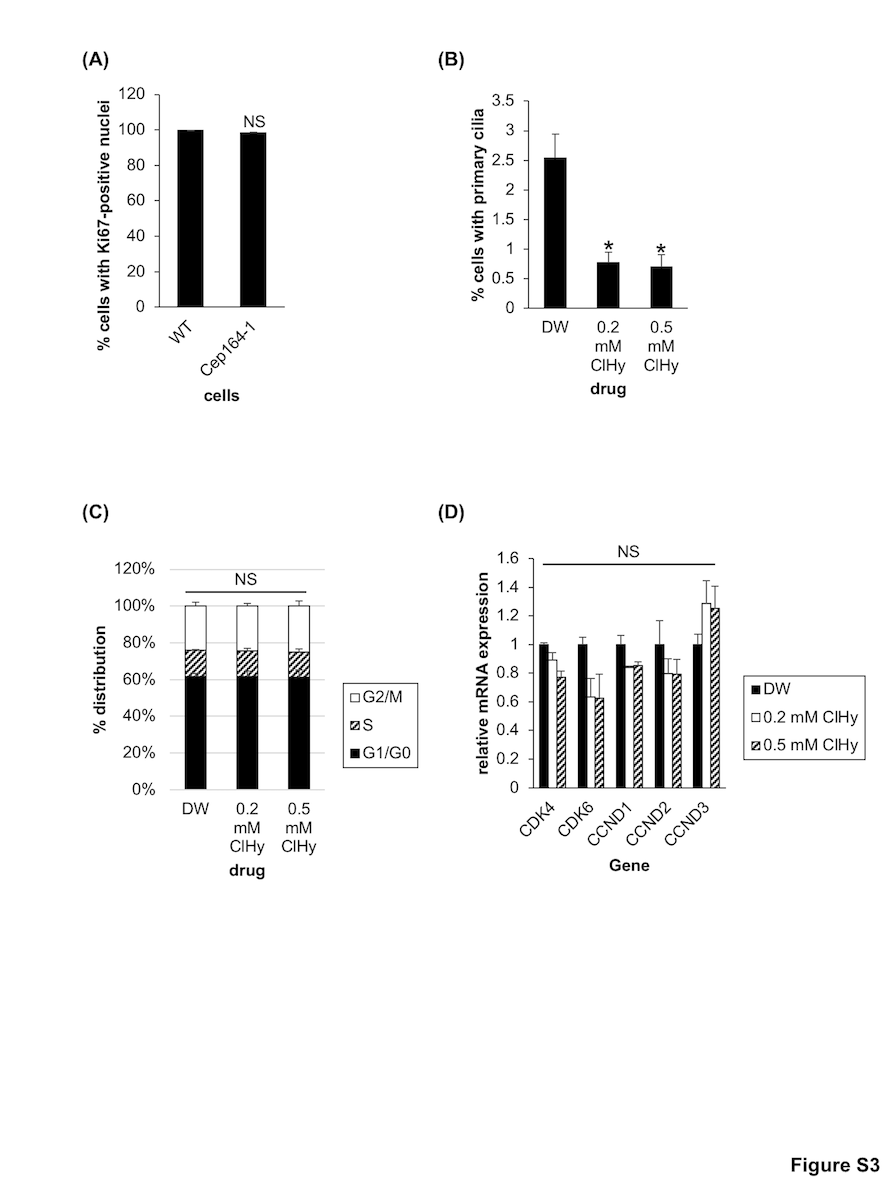

Supplement: Supplementary file 7 [file Image_3.tiff]

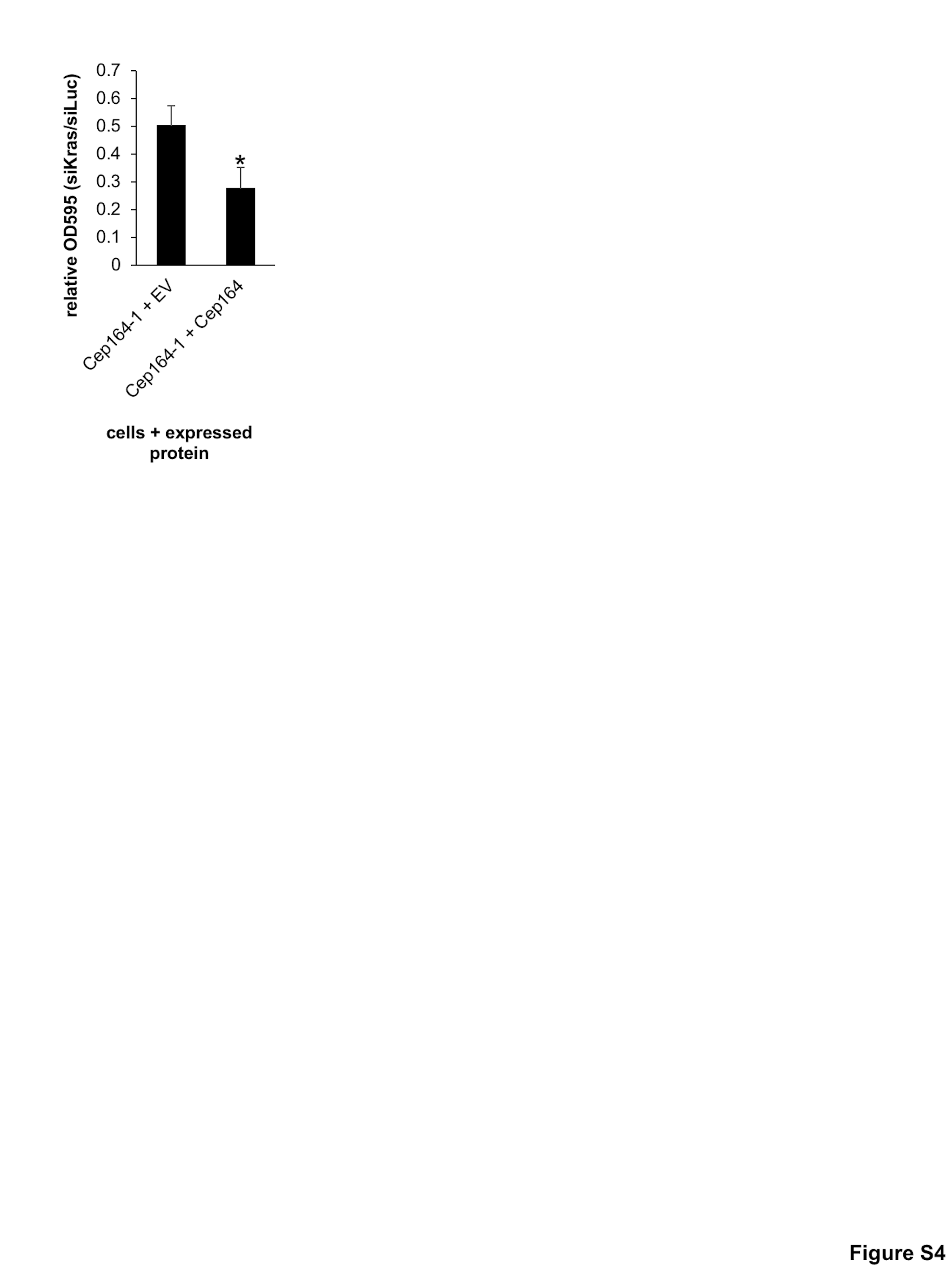

Supplement: Supplementary file 8 [file Image_4.TIFF]

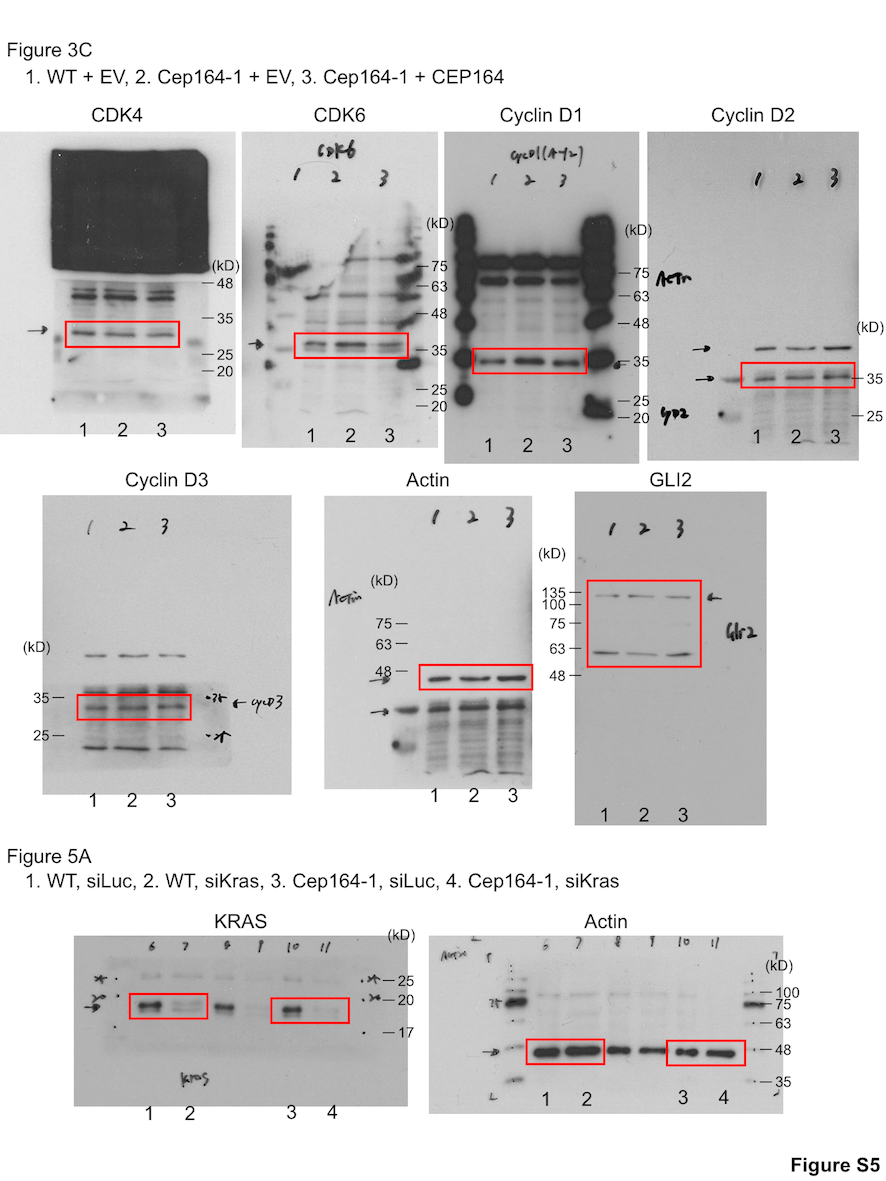

Supplement: Supplementary file 9 [file Image_5.tiff]
